# Alcohol Abuse and Suicide Attempt in Iran: *A Case-Crossover Study*

**DOI:** 10.5539/gjhs.v8n7p58

**Published:** 2015-11-03

**Authors:** Behrooz Ghanbari, Seyed Kazem Malakouti, Marzieh Nojomi, Diego De Leo, Khalid Saeed

**Affiliations:** 1 Mental Health Research Center (MHRC), Tehran Institute of Psychiatry, School of behavioral sciences and mental health, Iran University of Medical Sciences (IUMS), Tehran, Iran; 2 Department of Community Medicine, School of Medicine, Iran University of Medical Sciences (IUMS), Tehran, Iran; 3 Australian Institute for Suicide Research and Prevention, National Centre of Excellence in Suicide Prevention, WHO Collaborating Centre for Research and Training in Suicide Prevention, Life Promotion Clinic, Australia; 4 Mental Health and Substance Abuse unit, Department of Non-Communicable Diseases and Mental Health, World Health Organization, Regional Office for the Eastern Mediterranean, Australia

**Keywords:** alcohol abuse, case-crossover, suicide attempt

## Abstract

Alcohol use and its disorders are associated with increased risk of suicidal behaviors Research has shown that 6-8% of those who use alcohol have a history of suicide attempt. Given the prohibition of alcohol use legally, the increased alcohol consumption, and the lack of strong evidence in favor of its use associated with suicide in Iran, this study was conducted to determine the link between suicide attempt and alcohol abuse. The case-crossover method was used in this research. Out of 305 referrals to the emergency room due to a suicide attempt, 100 reported drinking alcohol up to six hours before their attempt. Paired Matching and Usual Frequency were employed to analyze the data with STATA 12.0. The probability of attempting suicide up to six hours after drinking alcohol appeared increased by 27 times (95% CI: 8.1-60.4). Separate analysis for each of these hours from the first to the sixth hour after alcohol use was also performed. Fifty percent of attempted suicides happened one hour after alcohol use. Relative risk for the first and second hour was 10% and 5% respectively. Alcohol use is a strong proximal risk factor for attempted suicide among Iranian subjects. Prevention of alcohol use should be considered in setting up of the national Suicide attempt prevention program.

## 1. Introduction

### 1.1 Introduce the Problem

Suicide is an important public health issue (Ghanbari, Malakouti, Nojomi, Alavi, & Khaleghparast, 2015). Rates of suicide in the world, and mainly in developing countries, have risen by 60% in the past 50 years (Evidence, Team, & Project, 2006). Suicide is the third leading cause of death in the 15 to 44-year-old age group in some countries (Hawton & van Heeringen, 2009). Despite the low rate of suicide in Eastern countries, because of their large population, suicides are the biggest (Evidence et al., 2006).

In Iran, suicide is the fifth leading cause of death (Morovatdar, Moradi Lakeh, Malakouti, & Nojomi, 2013), and suicide rates have shown an upward trend over the last three decades (Shojaei, Shamsiani, Moradi, Alaedini, & Khademi, 2012). Based on a report published by the Iranian Ministry of Health, the Disability Adjusted Life Years (DALY) for suicides and self-harming behaviors in the country in 2003 was 206.2 per 100 000 people (Hajebi et al., 2011). Suicide is a multi-factorial phenomenon influenced by biological, psychological, and social factors (Panaghi, 2010). Alcohol use and its disorders are associated with increased risk of suicidal behaviors (Cherpitel, Borges, & Wilcox, 2004). Research has shown that 6-8% of those who use alcohol have a history of suicide attempt (Cherpitel et al., 2004). Moreover, international research has showed a clear relationship between per capita alcohol consumption and suicide rates. When access to alcohol was restricted in Russia, suicide rates sharply declined (Cherpitel et al., 2004).

The acute effects of alcohol consumption can be measured by determining blood alcohol levels in weight per volume (such as mg/dl). These acute effects of alcohol become more severe when greater alcohol is consumed (Borges et al., 2006). Forensic Pharmacokinetics show Fifteen to twenty minutes after consumption, alcohol reaches the concentration in the blood that affects the brain. And it reaches peak time after 30 to 90 minutes (Bezchlibnyk-Butler, Jeffries, Procyshyn, & Virani, 2013). Pharmacodynamics of ethanol show that alcohol drinking influences the behavior and the actions of an individual, produces energy 7.1 kcal per g (1 gram of alcohol consumption), effects on Central Nervous System (CNS) as a psychotropic drug and Impaires the performance and behavior (Traversy & Chaput, 2015). Consumption of a standard drink (14 grams or about 18 milliliters of ethanol) raises blood alcohol level by 0.0-0.5% on average. Blood alcohol level decreases by about 0.015% per hour on average after stop drinking. Blood alcohol concentrations of over 0.20% represent serious poisoning, and concentrations of 0.35-0.40% can be potentially lethal (Dawson, Hingson, & Grant, 2011).

### 1.2 Objective

Considering the increased alcohol use (Cherpitel et al., 2004) and the lack of strong evidence of its association with Suicide attempt in Iran, this study designed to investigate relationship between suicide attempt and acute effects of alcohol use.

## 2. Methods

### 2.1 Study Design

The case-crossover method was used (Maclure & Mittleman, 2000). This design is useful when the risk factor/exposure is transient, short-term exposures on the risk of acute events (Jaakkola, 2003). This strategy, an individual serves as his or her own control in studying the effect of a transient factor (acute alcohol consumption) on the risk of an acute event (suicidal attempt). In this design was developed to circumvent previous limitations in the selection of proper control subjects in traditional case-control studies (Cherpitel et al., 2004). This study conducted on 305 people who attempted suicides and were referred to the Baharloo Hospital in Tehran from June to November 2013. We used sample size formula (1 - ß or power =90, α=0.005). The study designed to investigate the effects of using 120 cc of alcohol (equivalent to 2 glasses Aragh Sagi, Note 1) in risk period (six hours before suicide attempt) on the risk of attempting suicide (resulting in hospitalization).

Using 120 cc of alcohol represent blood alcohol concentrations of over 0.20% and results to Lethargy, confusion, stupor and coma (Dawson et al., 2011). Six-hour duration before attempt considered as a risk period because after this period the detrimental side effect of alcohol on the mentality will be erased enough which does not affect the consciousness of the individual (Bagge, 2010).

### 2.2 Inclusion & Exclusion Criteria

The inclusion criteria were:

1) History of drinking at least 120cc of alcohol in the six hours before the attempt.
2) Positive laboratory test for alcohol: Positive blood alcohol content (Berrouiguet, Gravey, Le Galudec, Alavi, & Walter, 2014). (Cut of point = concentrations of 0.20%).

The exclusion criteria were mental retardation and refusal to participate in the study.

### 2.3 Instruments

1) A demographic questionnaire including age, gender, marital status, occupation, education level, housing facilities, whether the patients had caregivers or someone who accompanied them, and the method of attempting suicide. These variables, the frequency of daily, weekly, monthly, and yearly use of alcohol, whether, regular or irregular and its use on the day suicide was attempt and later in previous attempts.
2) An alcohol questionnaire (adapted from the WHO questionnaire on alcohol and substance abuse of the SUPREMISS study) (Bertolote et al., 2005) including hours in which alcohol was consumed before the attempt, the amount and frequency of alcohol consumption and the disorders resulting from alcohol use. The questionnaire included this question: “Have you drank at least 120 cc of alcohol (2 glasses) before the attempt? And if yes, how many hours before attempt did you drink the alcohol?”
3) Blood Alcohol Content test (Hastedt, Herre, Pragst, Rothe, & Hartwig, 2012) was conducted using the gas chromatography method employing a Varian-cp model made in the U.S.A. The sensitivity of this test investigated in various studies and it is considered a golden standard compared to other tests measuring blood alcohol content (Hastedt et al., 2012; Zheng, Beck, & Helander, 2011). Those with blood alcohol more than 0.20%, which is equivalent to 120 cc alcohol consumption (equivalent to 2 glasses Aragh Sagi), are usually considered as alcohol positive (Hejazi, Timcheh harir, & vahdati, 2008).

### 2.4 Data Collection and Analysis

Interviews were conducted at the earliest opportunity following hospitalization and after receiving written consent from the participants. This study was to evaluate the effects of using at least 120 cc of alcohol, about 4 drinks (two glasses), on the risk of attempting suicide.

The risk period was considered to be 6 hours (Cherpitel et al., 2004) and the rest in the non-risk group. Then a separate analysis was performed to estimate the riskiest period for attempting suicide after alcohol use for each of the hours within the six hours after alcohol consumption. Paired matching and usual frequency models were employed for the analysis. In the first procedure, the individual was asked when and how much alcohol he/she used before attempting suicide. The conditional logistic regression model was employed to analyze this variable, and gender and age were then entered the model. In the second procedure, the frequency of alcohol use in the six hours before suicide was analyzed (at every hour); Odds ratio with 95% confidence interval was reported as an index of association. The data were analyzed with STATA 12.0.

### 2.5 Ethical Considerations

To observe ethical issues, the collected information was kept secret and, before the interviews, written consent was obtained from all participants. This study was approved by the ethics committee of the Iran University of Medical Sciences (number 2345).

## 3. Results

**Figure 1:**
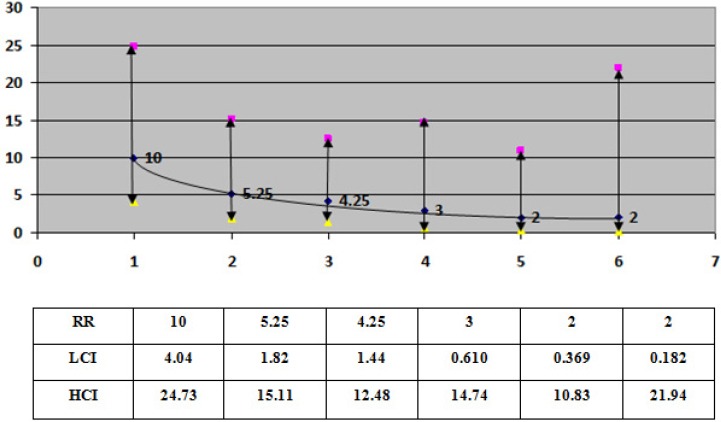
Relative risks for attempt suicide by time of alcohol use

Of the 352 people who had attempted suicide and were selected for interviews, two did not meet the criteria to enter the study and 45 refused to take part in the research. Of the remaining 305, 100 reported using alcohol 6 hours before attempting suicide. The average age of the participants was 26.5, with the standard deviation of 7.3. Considering only the subjects with alcohol abuse (n=100), the results showed 50% and 21% of the subjects who abused alcohol attempted suicide in the first and second hours. The prevalence of alcohol use during the risk period was greater in males compared to the females, and higher in singles compared to the married. Moreover, the amount of alcohol abused was greater during the weekend and by unemployed individuals (Tables 1-2). Most attempted suicide happened in the first (16%) and second hour (6%) while and the least frequent were in the sixth hour after alcohol use (Table 2). The higher RR was for the first hour of alcohol use before attempting suicide. Results are shown in Figure 1.

**Table 1:** Characteristics of subjects with and without alcohol abuse (six hours before attempt) (n=305)

Characteristics of subjects	abused alcohol in risk period (n=100)	not abused alcohol in risk period (n=205)	P valuea
**Sex**	n (%)	n (%)	

Male	52(52)	49(23.9)	0.001
Female	48(48)	156(76.1)	

**Education**			

No education & Elementary	9(9)	11(5.4)	0.438
Middle	16(16)	27(13.2)	
High school	55(55)	125(61)	
College or more	20(20)	42 (20.4)	

**Marital Status**			

Married	21(21)	99(48.3)	0.001
**Widow**	10(10)	10(4.8)	
single	69(69)	96(46.9)	

**Job Status**			

Unemployed	30(30)	51(24.9)	0.001
Housekeeper	5(5)	60(29.3)
Workless	55(55)	74(36.1)
Student	10(10)	20(9.8)

**History of Suicide attempt**			

Yes	51(51)	81(39.5)	0.029
No	49(49)	124(60.5)	

**Day of Suicide attempt**			

Sat	14(14)	27(13.2)	0.98
Sun	15(15)	28(13.7)	
Mon	13(13)	24(11.7)	
Tue	9(9)	40(19.5)	
Wed	9(9)	17(8.3)	
Thu	20(20)	20(9.8)	
Fri	20(20)	49(23.9)	

**Table 2:** Amount and frequency of alcohol consumption/abuse (n=305)

Time of drink (before Suicide attempt)	Male N (%)	Female N (%)	Total N (%)	*P* valuea
1 h	28(27.7)	22(10.8)	50(16)*	0.751
2 h	11(10.9)	10(4.9)	21(6)	
3 h	8(7.9)	9(4.4)	17(5)	
4 h	3(3)	3(1.5)	6(1)	
5 h	2(2)	2(1)	4(1)	
6 h	0(0)	2(1)	2(.5)	

No drink@	49(48.5)	156(76.5)	205(70.5)
No drink lifetime	45(44.1)	155(76.3)	200(65.6)	0.001
Drink<120cc	3(3)	2(1.1)	5(1.6)	
1- 3 times a year	5(4.9)	0(0)	5(1.6)	
4- 6 times a year	4(3.9)	2(1.1)	6(2)	

Monthly	22(21.5)	23(11.3)	45(14.7)	
Weekly	11(10.9)	15(7.3)	26(8.6)	
Daily	12(11.7)	6(2.9)	18(5.9)

* Considering only the 100 cases with alcohol abuse in six hours before attempt, it is showing that retrospectively from first to sixth hour, the prevalence of attempters were 50, 21, 17, 6, 4, 2 percent.

@ No drink in this study means no history of 4 drink in six hours before attempt and the blood sample is negative for BAC test The relative risks for every hour of risk period were evaluated.

Results obtained from the conditional logistic regression model for the chances of attempting suicide during the risk period compared to the non-risk period showed that the risk of attempted suicide was 27 times greater in the risk period. Furthermore, the risk of Suicide attempt was correlated with the mutual effects of age and sex. Table 3 shows results obtained from the model.

**Table 3:** Conditional logistic regression model showing odds ratio of some variables to Suicide attempt

Group	Odds Ratio	Std. Err	*P* value	Conf. Interval	-95%
**Alcohol**	21.902	11.219	*<* 0.001	8.0253	59.776

**Alcohol**	23.332	12.185	*<* 0.001	8.383	64.939
**Gender**	2.835	0.732	0.279	0.428	0.749

**Alcohol**	26.116	14.625	< 0.001	8.714898	78.267
**Age**	0.122	0.082	0.117	0.971	0.296

**Alcohol**	22.451	11.575	< 0.001	8.173	61.672
**Marital Status**	0.752	0.31	-0.691	0.335	1.689019

**Alcohol**	22.119	11.361	< 0.001	8.082	60.532
**Education**	0.905	0.223	0.688	0.558	1.468

**Alcohol**	27.463	15.83987	< 0.001	8.868	85.053
**Gender**	2.322	5.117	0.702	0.03	174.255
**Age**	1.09	0.422	0.824	0.509	2.331
**Marital Status**	1.13	2.247	0.951	0.023	55.595
**Education**	0.8365	0.799	0.852	0.128	5.447

## 4. Discussion

The main finding of this study showed that the alcohol abuse as a proximal stressor would increase the risk of attempt suicide by 27 times. Most attempts of suicide will occur in the first and second hour from alcohol abuse. The relative risk for the first and second hour is 10 and 5, which will decrease smoothly toward to the end of risk period.

The frequency of suicide attempts in the risk period for the first and second hours after alcohol use was up to 50 person (16%) and 21 (6%) of the study sample. In our study, the relationship between gender and age and suicide during the risk period was also investigated, but no relationship was found.

In this study, as a clinical sample, it was shown that about 33% of the participants used alcohol intermittently throughout their lifetime. As for frequency of alcohol use, our study showed that among the study sample (n=305) 14.7% consumed it on a monthly basis and a 14.5% on a weekly/daily basis.

About this, in the study conducted by Attar et al. (Attar, Afkham Ebrahimi, & Nasr Esfahani, 2004) on patients of Teheran hospital because of reasons except attempted suicide, it was reported that 9.6% of them used alcohol (Attar et al., 2004). Studies have shown the ratio of male to female users of alcohol is 1.3 (Sadock, Sadock, & Ruiz, 2000). This result conforms to those we have found and to those from other researches carried out in Iran (Allen et al., 1998). The lower frequency of alcohol use in women may rely on various reasons, possibly cultural, social, and religious.

The average age of participants in our study was 26.5 (SD=7.3). In research carried out by Attar (Attar et al., 2004), and by O’Farrell (O’Farrell, Allwright, Downey, Bedford, & Howell, 2004), most alcohol users were in the 15 to 29-year-old age group, and many those who consumed alcohol were single and unemployed, which conforms to the results of research carried out by Cherpitel (Cherpitel et al., 2004) and Morovatdar (Morovatdar, Moradi-Lakeh, Malakouti, & Nojomi, 2013). So, this age range may be considered as an important risk factor for alcohol abuse and suicide attempt.

Studies conducted by Cherpitel in the general population in the United States showed that 15% of people were alcohol abuser throughout their lifetime, and about 10% were dependent on it (Cherpitel et al., 2004).

Various studies carried out worldwide have pointed to alcohol as a risk factor for attempting suicide (Beautrais, 2000; Khan, Leventhal, Khan, & Brown, 2002; Kolves, Värnik, Tooding, & Wasserman, 2006; S. Malakouti et al., 2008; Morovatdar, Moradi Lakeh, et al., 2013; Pompili et al., 2010; Ramstedt, 2001; Leo Sher, 2005; L Sher, 2006). The research results showed that alcohol abuse resulting in death by suicide is about 10% and alcohol dependency in people who attempted suicide is close to 50% (Kolves et al., 2006).

A meta-analysis of cohort studies showed that heavy alcohol consumers have a five-fold higher risk of suicide than occasional drinkers (Harris & Barraclough, 1997). The mechanism of alcohol and suicide behaviors discussed by Pompili et al (Pompili et al., 2010) and Martinotti et al (Martinotti, Lupi, Santacroce, & Di Giannantonio, 2014). Comparing to suicide rate of general population, alcoholism could increase the risk of dying by suicide by up to 51 times (Suokas & Lönnqvist, 1995). One of main suicide prevention strategies is to reduce alcohol consumption (Mann et al., 2005).

Similar data are also emerging from developing countries. Although alcohol consumption has fallen in most developed countries since 1980, it has been raising in developing countries, with young people increasingly more exposed to misuse alcohol (Friel, Nic Gabhainn, & Kelleher, 1999; OECD, 2005). Population-based study in India showed that alcoholism associates significantly with suicide (Vijayakumar, 2004; Vijayakumar & Rajkumar, 1999).

In spite of convincing evidence of the relation between alcohol abuse and suicide, the relationship of alcohol drinking and suicide attempt has yet to be confirmed. The National Co-morbidity Survey (NCS) found no causal relationship between alcohol drinking and suicide attempts in adolescents (Chatterji, Dave, Kaestner, & Markowitz, 2004).

This may imply that only heavy users with alcohol dependent disorders are at risk of attempting suicide or dying by it. However, there is evidence showing that the impulsivity, disinhibit ion and misjudgment that may be caused by alcohol misuse can predispose to any kind of suicide behaviors (Conner & Duberstein, 2004). Study conducted on Indian patients with suicide attempt, showed that 17% of survivors had a history of alcohol intake prior to their suicide attempt (Bhattacharjee et al., 2012).

Regarding Middle East and Islamic countries, a limited number of studies has been conducted on suicide behaviors so far (S. K. Malakouti et al., 2015; Rezaeian, 2008) and the cultural and ideological basis has been challenged by Rezaeian (2008). One study carried in Iran revealed the odds of lifetime alcohol misuse for suicide attempt is 3.8 (Nojomi, Malakouti, Bolhari, Posht Mashadi, & Asghar Zadeh Amin, 2007).

In this regard the result of this study conducting with case-crossover design could help us to understand the relationship of alcohol drink, as a proximal precipitating factor for suicide attempt. A six-hour period before attempt has been considered as risky since after this timeframe the detrimental effects of alcohol would be unable to significantly affect the psycho-motor performances of the individual.

Results of research by Bagge showed more than 40% of those who attempted suicide reported using alcohol on the day of their attempt (Bagge, 2010).

In studies carried out in the US, the risk of attempt within six hours from alcohol intake was 5 times higher, and 68% of suicide attempt cases happened in the risky period after using alcohol (Bagge et al., 2013; Borges, Cherpitel, & Mittleman, 2004; Borges, Cherpitel, Macdonald, et al., 2004).

In the study conducted by Borges (Borges & Rosovsky, 2013) in emergency units of 8 hospitals using the case-control method, the risk of attempting suicide in people dependent on alcohol was up to 31 times higher, while it was up to two times in people who were not dependent on alcohol but had used alcohol before suicide attempt.

## 5. Conclusion

Suicide as complicated behaviors of human has multiple an interactive risk factors. This study showed that alcohol use (at least 120cc with positive result of BAC test), regardless of dependency or occasional consumption, plays an important and significant role as proximal factor for attempted suicide. In spite of alcohol use being forbidden by law and religious beliefs in some countries, given its increasing use in developing countries, alcohol use/disorders have to be included in suicide prevention programs.

## References

[ref1] Allen L. M, Nelson C. J, Rouhbakhsh P, Scifres S. L, Greene R. L, Kordinak S. T, Morse R. M (1998). Gender differences in factor structure of the Self-Administered Alcoholism Screening Test. Journal of clinical psychology.

[ref2] Attar H, Afkham Ebrahimi A, Nasr Esfahani M (2004). Alcohol Use in Hospitalized Patients at Hazrat-e-Rasoul Hospital. Iranian Journal of Psychiatry and Clinical Psychology.

[ref3] Bagge C. L (2010). Acute Alcohol Use and Suicide Attempts. Context.

[ref4] Bagge C. L, Lee H.-J, Schumacher J. A, Gratz K. L, Krull J. L, Holloman G (2013). Alcohol as an acute risk factor for recent suicide attempts: A case-crossover analysis. Journal of studies on alcohol and drugs.

[ref5] Beautrais A. L (2000). Risk factors for suicide and attempted suicide among young people. Australian and New Zealand Journal of Psychiatry.

[ref6] Berrouiguet S, Gravey M, Le Galudec M, Alavi Z, Walter M (2014). Post-acute crisis text messaging outreach for suicide prevention: A pilot study. Psychiatry research.

[ref7] Bertolote J. M, Fleischmann A, De Leo D, Bolhari J, Botega N, De Silva D, Värnik A (2005). Suicide attempts, plans, and ideation in culturally diverse sites: The WHO SUPRE-MISS community survey. Psychological Medicine.

[ref8] Bezchlibnyk-Butler K. Z, Jeffries J. J, Procyshyn R. M, Virani A. S (2013). Clinical handbook of psychotropic drugs: Hogrefe Verlag.

[ref9] Bhattacharjee S, Bhattacharya A, Thakurta R. G, Ray P, Singh O. P, Sen S (2012). Putative effect of alcohol on suicide attempters: An evaluative study in a tertiary medical college. Indian journal of psychological medicine.

[ref10] Borges G, Cherpitel C, Mittleman M (2004). Risk of injury after alcohol consumption: A case-crossover study in the emergency department. Social science & medicine.

[ref11] Borges G, Cherpitel C, Orozco R, Bond J, Ye Y, Macdonald S, Poznyak V (2006). Multicentre study of acute alcohol use and non-fatal injuries: Data from the WHO collaborative study on alcohol and injuries. Bulletin of the World Health Organization.

[ref12] Borges G, Cherpitel C. J, Macdonald S, Giesbrecht N, Stockwell T, Wilcox H. C (2004). A case-crossover study of acute alcohol use and suicide attempt. Journal of Studies on Alcohol and Drugs.

[ref13] Borges G, Rosovsky H (2013). Suicide attempts and alcohol consumption in an emergency room sample.

[ref14] Chatterji P, Dave D, Kaestner R, Markowitz S (2004). Alcohol abuse and suicide attempts among youth. Economics & Human Biology.

[ref15] Cherpitel C. J, Borges G. L. G, Wilcox H. C (2004). Acute alcohol use and suicidal behavior: A review of the literature. Alcoholism: Clinical and Experimental Research.

[ref16] Conner K. R, Duberstein P. R (2004). Predisposing and precipitating factors for suicide among alcoholics: empirical review and conceptual integration. Alcoholism: Clinical and Experimental Research.

[ref17] Dawson D. A, Hingson R. W, Grant B. F (2011). Epidemiology of Alcohol Use, Abuse and Dependence. Textbook in Psychiatric Epidemiology.

[ref18] Evidence W. H. O. M. H, Team R, Project D. C. P (2006). Disease control priorities related to mental, neurological, developmental and substance abuse disorders.

[ref19] Friel S, Nic Gabhainn S, Kelleher C (1999). The national health and lifestyle surveys.

[ref20] Ghanbari B, Malakouti S, Nojomi M, Alavi K, Khaleghparast S (2015). Suicide Prevention and Follow-Up Services: A Narrative Review. Global journal of health science.

[ref21] Hajebi A, Ahmadzad Asl M, Zaman M, Naserbakht M, Mohammadi N, Davoudi F, Saberi Zafarghandi M (2011). Designing a Registration System for Suicide in Iran. Iranian Journal of Psychiatry and Clinical Psychology.

[ref22] Harris E. C, Barraclough B (1997). Suicide as an outcome for mental disorders. A meta-analysis. The British Journal of Psychiatry.

[ref23] Hastedt M, Herre S, Pragst F, Rothe M, Hartwig S (2012). Workplace alcohol testing program by combined use of ethyl glucuronide and fatty acid ethyl esters in hair. Alcohol and alcoholism.

[ref24] Hawton K, van Heeringen K (2009). Suicide. Lancet.

[ref25] Hejazi A, Timcheh Harir A, Vahdati N (2008). A comparison of the effective inhalation detector with GC in alcoholism in Khorasan Razavi Legal Medicine Center. SJFM.

[ref26] Jaakkola J (2003). Case-crossover design in air pollution epidemiology. European Respiratory Journal.

[ref27] Khan A, Leventhal R. M, Khan S, Brown W. A (2002). Suicide risk in patients with anxiety disorders: a meta-analysis of the FDA database. Journal of affective disorders.

[ref28] Kolves K, Värnik A, Tooding L.-M, Wasserman D (2006). The role of alcohol in suicide: a case-control psychological autopsy study. Psychological medicine.

[ref29] Maclure M, Mittleman M. A (2000). Should we use a case-crossover design?. Annual review of public health.

[ref30] Malakouti S, Nojomi M, Posht Mashhadi M, Hakim Shoshtari M, Asgharzadeh Amin S, Bolhari J, Moshirpour S (2008). The study of suicidal behaviors rates in the community sample of Karaj city in 2005. Scientific Journal of Hamadam University of Medical Sciences and Health Services.

[ref31] Malakouti S. K, Davoudi F, Khalid S, Asl M. A, Khan M. M, Alirezaei N, DeLeo D (2015). The Epidemiology of Suicide Behaviors among the Countries of the Eastern Mediterranean Region of WHO: A Systematic Review. Acta Medica Iranica.

[ref32] Mann J. J, Apter A, Bertolote J, Beautrais A, Currier D, Haas A, Marusic A (2005). Suicide prevention strategies: A systematic review. Jama.

[ref33] Martinotti G, Lupi M, Santacroce R, Di Giannantonio M (2014). Alcohol use disorders and suicidal behaviour: a clinical review of studies in developed and developing countries. Research and Advances in Psychiatry.

[ref34] Morovatdar N, Moradi-Lakeh M, Malakouti S. K, Nojomi M (2013). Most Common Methods of Suicide in Eastern Mediterranean Region of WHO: A Systematic Review and Meta-Analysis. Archives of Suicide Research.

[ref35] Morovatdar N, Moradi Lakeh M, Malakouti S. K, Nojomi M (2013). Frequency of Methods of Suicide in Eastern Mediterranean Region (EMRO) of WHO: A Systematic Review. Iranian Journal of Psychiatry and Clinical Psychology.

[ref36] Nojomi M, Malakouti S. K, Bolhari J, Posht Mashadi M, Asghar Zadeh Amin S (2007). Predicting Factors of Suicide Attempts in Karaj General Population. Iranian Journal of Psychiatry and Clinical Psychology.

[ref37] O’Farrell A, Allwright S, Downey J, Bedford D, Howell F (2004). The burden of alcohol misuse on emergency in-patient hospital admissions among residents from a health board region in Ireland. Addiction.

[ref38] OECD W (2005). Organisation for Economic Development and Co-operation: OECD Health Data 2005: Statistics and Indicators for 30 Countries.

[ref39] Panaghi L, Ahmadabadi Z, Peyravi H, Abou A. F (2010). Suicide trend in University students during 2003 to 2008.

[ref40] Pompili M, Serafini G, Innamorati M, Dominici G, Ferracuti S, Kotzalidis G. D, Tatarelli R (2010). Suicidal behavior and alcohol abuse. International journal of environmental research and public health.

[ref41] Ramstedt M (2001). Alcohol and suicide in 14 European countries. Addiction.

[ref42] Rezaeian M (2008). Islam and suicide: A short personal communication. OMEGA--Journal of Death and Dying.

[ref43] Sadock B, Sadock V, Ruiz P (2000). Comprehensive textbook of psychiatry.

[ref44] Sher L (2005). Alcohol use and suicide rates. Medical hypotheses.

[ref45] Sher L (2006). Alcohol consumption and suicide. Qjm.

[ref46] Shojaei A, Shamsiani H, Moradi S, Alaedini F, Khademi A (2012). The Study of Successful Cases of Suicide Commitment Referred to Iran Legal Medicine Organization in 2010. SJFM.

[ref47] Suokas J, Lönnqvist J (1995). Suicide attempts in which alcohol is involved: A special group in general hospital emergency rooms. Acta Psychiatrica Scandinavica.

[ref48] Traversy G, Chaput J.-P (2015). Alcohol consumption and obesity: an update. Current obesity reports.

[ref49] Vijayakumar L (2004). Suicide prevention: the urgent need in developing countries. World Psychiatry.

[ref50] Vijayakumar L, Rajkumar S (1999). Are risk factors for suicide universal? A case-control study in India. Acta psychiatrica scandinavica.

[ref51] Zheng Y, Beck O, Helander A (2011). Method development for routine liquid chromatography–mass spectrometry measurement of the alcohol biomarker phosphatidylethanol (PEth) in blood. Clinica Chimica Acta.

